# Prediction of Delirium in ICU Patients and Development of a CDSS Model Using Clinical Data Warehouse

**DOI:** 10.1093/geroni/igaf122.2321

**Published:** 2025-12-31

**Authors:** Sun Ju Kim

**Affiliations:** Chungnam National University Se-Jong Hospital, Se-Jong, Republic of Korea

## Abstract

Delirium in intensive care unit (ICU) patients is a critical condition associated with increased hospital stay, mortality, and healthcare costs. Using a Clinical Data Warehouse (CDW), key risk factors influencing the occurrence of delirium in ICU patients are identified and a Clinical Decision Support System (CDSS) for early prediction and prevention developed. From May 2015 to May 2024, a retrospective cohort study involving adult patients (≥18 years) admitted to the ICU of a tertiary hospital in Daejeon, South Korea was conducted. Patients who developed or did not develop delirium within five days of ICU admission were classified as the experimental and control group, respectively. Predictive models were developed and compared using machine learning techniques, including logistic regression, decision tree, and XGBoost. Demographics, biometric indicators, vital signs, and past medical history were key predictive variables. The decision tree model demonstrated high sensitivity, whereas the XGBoost model exhibited high accuracy (0.66) and specificity, thereby making it an effective tool for delirium prediction. Logistic regression provided easy interpretability but limitations were observed due to lower sensitivity (0.22). GCS-Verbal, GCS-Eye, age, blood pressure, and oxygen saturation were the most significant predictive variables. Accordingly, a CDSS was designed to proactively assess the risk of delirium in ICU patients. The performance of machine learning-based models for delirium prediction was compared and the feasibility of integrating these models into a CDSS explored. The proposed CDSS can facilitate early detection and intervention strategies for ICU delirium. Future research should focus on model optimization and real-time clinical implementation.

